# Supplementary material for: Production of an Active, Human Membrane Protein in Saccharomyces cerevisiae: Full-Length FICD
Source: Int J Mol Sci. 2022 Feb 23;23(5):2458. doi: 10.3390/ijms23052458 (PMC8910494; doi:10.3390/ijms23052458)
Supplement: Supplementary file 1 [file ijms-23-02458-s001.zip › ijms-1601876-supplementary.pdf]

Supplementary Materials

Production of an Active, Human Membrane Protein in *Saccharomyces cerevisiae*: Full-Length FICD

Minttu S. Virolainen, Cecilie L. Søltoft, Per A. Pedersen and Lars Ellgaard

Table S1. Primers used to generate the His8-GFP-TEV-FICD and His8-FICD constructs

Primer no.	Primer name	Sequence (5´-3´)
1	pEMBLyex4-His8-GFPFWD	ACACAAATACACACACTAAATTACCGGATCAATTCTAAGATAATTATGCATCACCATCACCATCACCATCACATGTCTAAAGGTGAAGAATTATTCACT
2	GFP-TEVREV	AAATTGACTTTGAAAATACAAATTTTCTTTGTACAATTCATCCATACCATGG
3	TEV-FICDFWD	GAAAATTTGTATTTTCAAAGTCAATTTATGATGCTAATACCTATGGCGTC
4	pEMBLyex4b- FICDREV	CTTCAATGCTATCATTTCCTTTGATATTGGATCATTTAGGGCTTAACAGGCAGAGTC
5	pEMBLyex4- His8-FICDFWD	ACACAAATACACACACTAAATTACCGGATCAATTCTAAGATAATTATGCATCATCACCACCACCATCATCACATGATGCTAATACCTATGGCGTC

Light blue; pEMBLyex4b vector sequence, magenta; Kozak sequence, yellow; start codon, purple; His8-tag sequence, green; GFP sequence, cyan; TEV-cleavage site, dark blue; FICD sequence, red; stop codon.

Table S2: Biophysical characteristics of the detergents used in the present study. CMC values were obtained from the suppliers Affymetrix and Glycon Biochemicals. Solubilisation screens were performed at a protein concentration of 2 mg/ml and detergent concentrations between 2 mg/ml and 6 mg/ml (see Materials and Methods) giving the indicated concentration ranges of detergent and times CMC values. The publicly available radius of gyration (R_g) values were taken from [1] and [2]. It was not possible to identify R_g values for all detergents.

Detergent abbreviation	CMC (mM)	Solubilisation concentration (mM)	Solubilisation conditions (times CMC)	R_g (Å)
MNG	0.01	2.0 – 6.0	200 – 600	-
DM	1.8	4.1 – 12.4	2.3 – 6.9	26.2
DDM	0.17	3.9 – 11.8	22.9 – 69.4	31.8
TDM	0.033	3.8 – 11.4	115 – 345	43.5
HDM	0.0006	3.5 – 10.6	5833 – 17667	-
OG	18	6.8 – 20.5	0.4 – 1.1	29.6
NG	6.5	6.5 – 19.6	1 – 3	34.2
PCCM	0.036	3.7 – 11.0	103 – 306	-
FC12	1.5	5.7 – 17.1	4 – 11	34.2
FC13	0.75	5.5 – 16.4	7 – 22	34.5
LDAO	1-2	8.7 – 26.2	9 – 26	18.8
CHS	-	1.1 – 3.3	-	-

CMC; critical micelle concentration, R_g; radius of gyration

Table S3. Primers used in construction of *E. coli* expression vectors

Primer no.	Primer name	Sequence (5′-3′)
6	FICD102$_{FWD}$	TAGAACCATGGCCAAATTAGAGGCCCGCGCAGCT
7	FICD458$_{REV}$	ATTAACGGATCCTTAAGGTTTTACTGGCAACGT
8	BiP T229A$_{FWD}$	GCGGTGGAGCCTTCGATGTGTCTCTTCTG
9	BiP T229A$_{REV}$	ACATCGAAGGTTCCACCGCCCAGGTCAAA
10	BiP V461F$_{FWD}$	CAGCCAACTTTTACAATCAAGGTCTATGAA
11	BiP V461F$_{REV}$	CTTGATTGTAAAAGTTGGCTGATTATCAGA

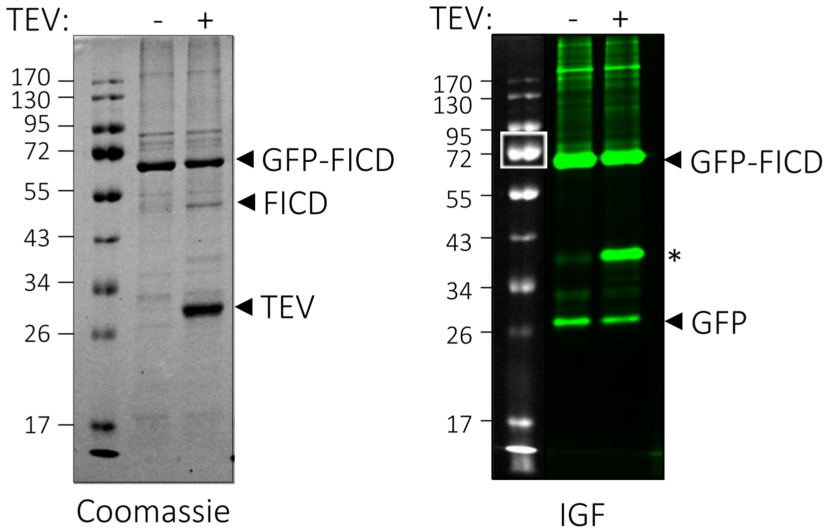

Figure S1. Inefficient TEV protease cleavage of GFP-FICD. **A)** SDS-PAGE analysis of IMAC purified GFP-FICD before (-) and after (+) TEV cleavage by Coomassie staining (left) and in-gel fluorescence (IGF, right). GFP-FICD was incubated with TEV protease in a 1:1 molar ratio overnight at 4°C. An unidentified cleavage product is marked by an asterisk (*).

References

1. Lipfert, J.; Columbus, L.; Chu, V.B.; Lesley, S.A.; Doniach, S. Size and Shape of Detergent Micelles Determined by Small-Angle X-ray Scattering. *J. Phys. Chem. B* **2007**, *111*, 12427–12438. https://doi.org/10.1021/jp073016l.
2. Oliver, R.C.; Lipfert, J.; Fox, D.A.; Lo, R.H.; Kim, J.J.; Doniach, S.; Columbus, L. Tuning micelle dimensions and properties with binary surfactant mixtures. *Langmuir* **2014**, *30*, 13353–13361. https://doi.org/10.1021/la503458n.
